# Case report: Recovery and sequential imaging of a patient with osmotic demyelination syndrome

**DOI:** 10.3389/fvets.2023.1146091

**Published:** 2023-04-28

**Authors:** Stephanie Harris, Adrien Dupanloup, Pen-Ting Liao, Tom Jukier

**Affiliations:** ^1^Department of Clinical Sciences, Auburn University College of Veterinary Medicine, Auburn, AL, United States; ^2^Veterinary Medical Teaching Hospital, School of Veterinary Medicine, University of California, Davis, Davis, CA, United States

**Keywords:** pontine myelinolysis, extrapontine demyelination, osmotic demyelinating syndrome, magnetic resonance imaging, dog, veterinary

## Abstract

A 4-year-old neutered-male Australian Shepherd was presented to an emergency and referral hospital for an acute onset of neurologic signs and abnormal mentation. Seven days prior, the patient had been diagnosed with hypoadrenocorticism and was treated accordingly at another hospital. Based on recent clinical history, the neurologic signs were consistent with thalamic and brainstem deficits and suspected to be caused by osmotic demyelination syndrome secondary to rapid correction of hyponatremia. A brain MRI confirmed lesions consistent with osmotic demyelination syndrome. The patient's clinical signs initially worsened, and he required intensive nursing care with multimodal sedation, close monitoring of electrolytes and tailored fluid therapy. The patient recovered and was discharged on day seven of hospitalization. Four and a half months later, re-evaluation of the patient showed complete resolution of the neurological deficits with a now unremarkable neurological exam, and follow-up MRI revealed still present, yet improved bilateral thalamic lesions. This is the first known veterinary case report of sequential brain imaging of a dog that has recovered from osmotic demyelination syndrome. In humans, patients can have evidence of near to full clinical recovery, yet imaging findings may still be abnormal several months after recovery. This report details similar imaging findings in a canine with improved clinical signs, despite persistent lesions on brain MRI. Prognosis of canines with osmotic demyelination syndrome may be better than previously perceived, despite the severity of clinical signs and brain lesions apparent on MRI.

## 1. Introduction

Osmotic demyelination syndrome (ODS), formerly referred to as “pontine and extrapontine myelinolysis,” is a condition first described in four individuals with alcoholism and malnourishment by Adams et al. (1). This condition was later associated with rapid correction of chronic hyponatremia and with this, a wide spectrum of symptoms have been described, including severe movement disorders, mental status changes, and acute death (2, 3). Histopathologically, degeneration of oligodendrocytes and myelin is evident, with the types of lesions varying from mild edema noted in myelin and intracellularly, to more severe lesions characterized by myelinolysis, degeneration of oligodendrocytes, and degeneration of axons and neuronal cell bodies, with the latter occurring at the center of the lesion (1, 4). There are infrequent reports in veterinary medicine describing ODS, both with and without recovery, with few documenting MRI lesions at time of diagnosis and post-mortem (5, 6). In human medicine, the prognosis was initially described to be grave. However, with the advent of advanced imaging, appropriate nursing care, sedation and regular monitoring of sodium levels, the prognosis may be more favorable with good recovery in more than half of cases (7).

## 2. Case description

A 4-year-old male castrated Australian Shepherd weighing 10.7 kg was presented to the emergency service of a veterinary teaching hospital for a rapidly worsening tetraparesis with deterioration of mental status of a 2-day duration. Seven days prior to presentation, the patient was treated at another veterinary hospital for gastrointestinal signs, where he was hospitalized and diagnosed with hypoadrenocorticism based on ACTH stimulation testing (pre- and post- cortisol levels were < 0.2 ug/dL). After 3 days of hospitalization, the patient clinically improved and was discharged with his first mineralocorticoid injection^1^ and 0.25 mg/kg/day of prednisone.^2^ No additional information was available at that time, other than the patient's sodium to potassium ratio had been low. Two days after discharge, the owner noted that the patient was becoming weak in his pelvic limbs, which seemed to worsen over the following day. Pelvic muscle fasciculations also developed and were most notable when attempting to eat or drink. At that point, the patient was referred to the authors' institution for further workup of progressive neurologic signs.

On presentation, the patient's general physical examination was within normal limits. On neurological examination, the patient vocalized intermittently, and his mentation was described as disoriented. A left head turn was present. He was non-ambulatory but could stand with support. The thoracic limbs showed increased extension, and a severe proprioceptive ataxia was noted when assisted to walk. Postural reactions (knuckling and hopping) were absent in the thoracic limbs and decreased in the pelvic limbs. Limb reflexes tested were normal. Cranial nerve assessment revealed absent menace response bilaterally, present but decreased palpebral reflex, decreased sensation of the face and pinnae, and symmetrically mild atrophy of muscles of mastication. Given these neurological findings, neurolocalization was considered to likely be multifocal, with involvement of the brainstem and thalamus. Due to these findings and patient history, the leading differential was ODS, with other differentials that were considered less likely being an inflammatory or infectious process.

The patient was admitted for further diagnostics, supportive care, and monitoring. A minimal database was obtained. The chemistry profile revealed abnormalities of a sodium of 154 mmol/L (142–151), a potassium of 6 mmol/L (3.6–4.9), chloride of 123 mmol/L (105–117), calcium of 2.25 mmol/L (2.4–2.99), along with an albumin of 0.3897 mmol/L (0.451–0.647) and a total protein at 5.24 g/dL (5.50–7.70). The urinalysis and CBC results were within the institution's normal reference intervals. The patient was placed on an isotonic balanced crystalloid^3^ at 32 ml/h and was administered dexamethasone SP^4^ IV at 0.05 mg/kg/day. Over the following 24 h, the patient's neurological status continued to progress where he became more agitated and vocal, and his tetraparesis worsened so that he could no longer stand without support. Due to severe agitation, he was administered butorphanol^5^ at 0.3 mg/kg IV, which did not change his clinical signs. He was then administered 0.5 mg/kg midazolam^6^ IV, which temporarily alleviated his signs. However, his agitation worsened again rapidly with severe continual vocalization that manifested as paroxysmal events, which motivated initiation of levetiracetam^7^ at 30 mg/kg IV q8h, for which an equivocal response was noted.

The following day, medical records from the patient's recent hospitalization were obtained, and it was noted that 9 days prior, the patient's initial serum sodium level was reported to be 106 mg/dL (144–160 mg/dL). During that hospitalization, the patient had received a 250 mL bolus of 0.9% NaCl^8^, followed by 60 ml/h over the following 3 days (a total of 2,590 mL). There was no record of sodium levels being checked until time of referral; therefore, it is unknown how quickly the sodium level had risen.

On day three of hospitalization, the patient's clinical signs continued to progress where his mentation and agitation worsened, and he developed difficulty with prehension of food. For sedation, he was placed on both CRI's of dexmedetomidine^9^ (1–5 mcg/kg/h) and midazolam (see text footnote 6; 0.1–0.5 mg/kg/h), which successfully limited his agitation to times of stimulation. The patient's electrolytes were monitored at minimum every 24 h, and his sodium had slightly increased from 154 to 155 mmol/L (142–151 mmol/L). The patient's potassium was noted to be decreasing from 4.2 mmol/L on presentation to 3.7 mmol/L (3.6–4.9 mmol/L). Therefore, spironolactone^10^ (12.5 mg PO q24h) was added as a potassium-sparing diuretic in attempt to prevent further increase in the sodium level. The isotonic balanced crystalloid (see text footnote 3) was also discontinued and a hypotonic solution^11^ was initiated at maintenance rate. Due to the patient's increased sedation and recumbency, a urinary catheter was placed to aid with nursing care and to allow monitoring of urine output.

**Figure 1:**
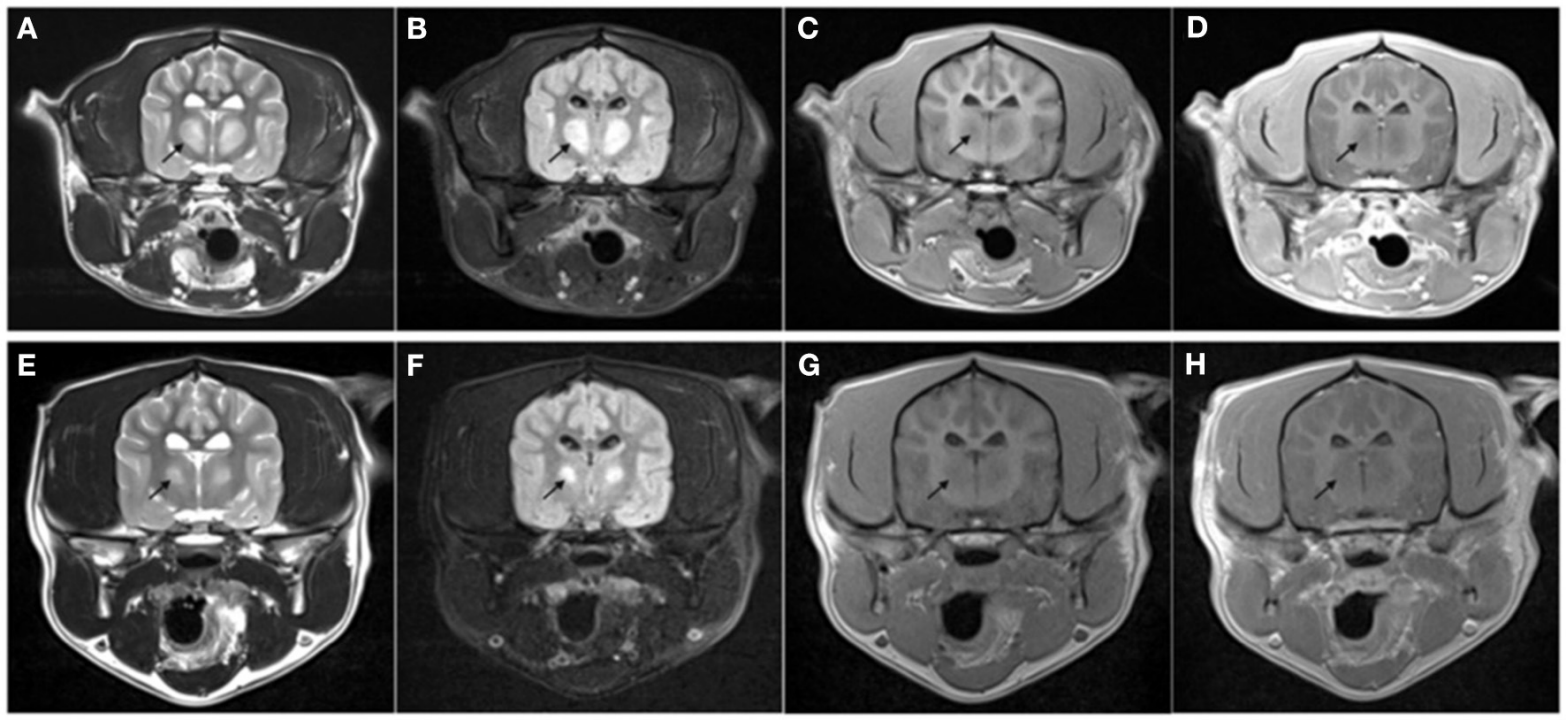
Transverse MRI images performed on day 4 of hospitalization **(A–D)** and at recheck 130 days later **(E–H)**. The images are at the level of the thalamus. T2-weighted **(A, E)**, T2 FLAIR **(B, F)**, T1-weighted pre- **(C, G)**, and post- **(D, H)** administration of intravenous gadolinium. Note the bilateral symmetric T2W and T2-FLAIR hyperintense [arrow in **(A, E)**], T1 hypointense non-contrast enhancing lesions present within the thalamus. The lesion was markedly reduced in size at follow-up MRI with the same MRI characteristics.

On day four of hospitalization due to continued progression of clinical signs, the patient was anesthetized for MRI and CSF analysis. MRI showed a bilaterally symmetric T2W/T2-FLAIR hyperintense/T1 hypointense non-contrast enhancing lesion within the thalamus and to a lesser extent the caudal aspect of the caudate nuclei, rostral cerebral peduncles, and claustrum (see Figure 1). DWI showed hyperintense signal in the corresponding lesions on B0 images, and mildly hypointense signal on the B1000 (Figure 3). The ADC map showed hyperintense signal in the same areas. These findings suggest lack of fluid restriction in the affected areas and would support vasogenic edema over cytotoxic edema. Given the patient's history MRI findings, progression, myelinolysis was considered the primary differential however, inflammatory/infectious process was also considered. Clinical pathologic review of CSF was cytologically unremarkable. Given the signalment at time of development of clinical signs, other demyelinating conditions were considered to be much less likely.

The findings were discussed with the owner and based on historical reports in both human and veterinary literature, a fair to guarded prognosis was given (2, 8, 9). The owner elected to continue supportive care and monitoring over the following days. A nasogastric tube was administered to facilitate enteral nutrition. The patient's midazolam (see text footnote 6) and dexmedetomidine (see text footnote 9) infusions were continued. For additional sedation, a ketamine^12^ CRI (0.1–0.5 mg/kg/h) was started, and a metoclopramide^13^ CRI (5 mg/kg/day) was initiated for promotility effects to decrease risk of aspiration pneumonia. Liquid enteral nutrition^14^ was initiated through his nasogastric tube.

On day five of hospitalization, the patient seemed more aware of his surroundings and appeared visual as witnessed by tracking movements. He was calmer and less hyperreactive. Physical therapy was initiated with range of motion and standing exercises. He continued to show marked improvement throughout the day and began to stand and ambulate independently. That evening he started to eat on his own. His urinary catheter, nasogastric tube and IV catheter were all removed. Metoclopramide (see text footnote 13) was discontinued, and the intravenous medications dexamethasone SP (see text footnote 4), ketamine (see text footnote 12), and dexmedetomidine (see text footnote 4) were discontinued and transitioned to oral prednisone (see text footnote 2; 0.5 mg/kg/day), amantadine^15^ (0.4 mg/kg/day), and trazodone^16^ (3–5 mg/kg/day), respectively.

The next day, the patient continued to improve. He was more ambulatory with improving ataxia and he exhibited less agitation. He was discharged on day seven with oral prednisone (see text footnote 2), amantadine (see text footnote 15), and trazodone (see text footnote 16). A recheck exam 1 week later revealed a normal neurological examination.

### 2.1. Follow up

**Figure 2:**
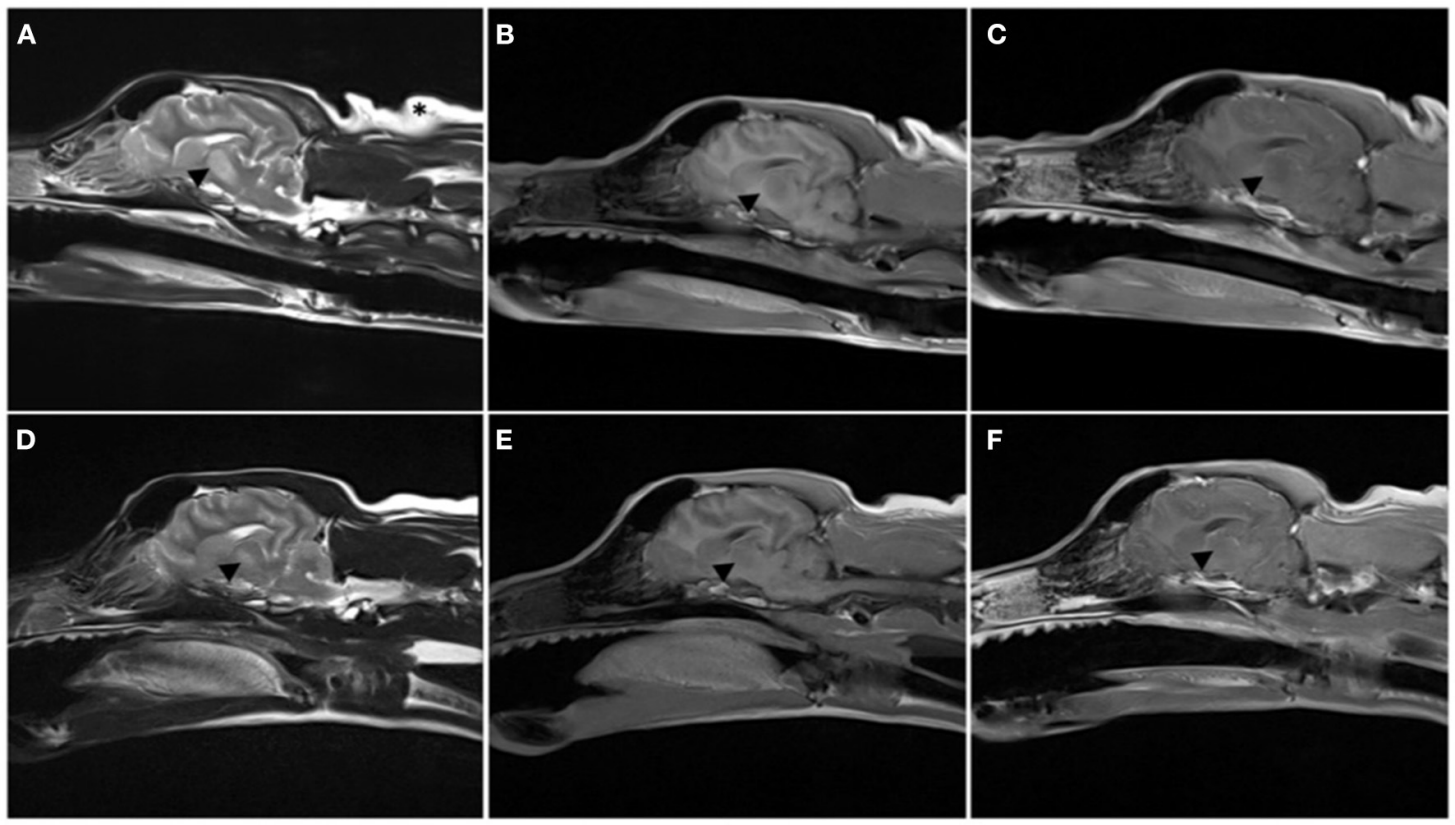
Parasagittal MRI images performed on day 4 of hospitalization **(A–C)** and at recheck 130 days later **(D–F)**. T2-weighted **(A, D)**, T1-weighted pre- **(B, E)**, and post- **(C, F)** administration of intravenous gadolinium. Note the T2W hyperintense lesion extending from the thalamus to the midbrain [arrowhead in **(A)**], T1 hypointense non contrast enhancing lesion is mostly present within the thalamus. The lesion was markedly reduced in size at follow-up MRI, only present in the thalamus, and with the same MRI characteristics.

**Figure 3:**
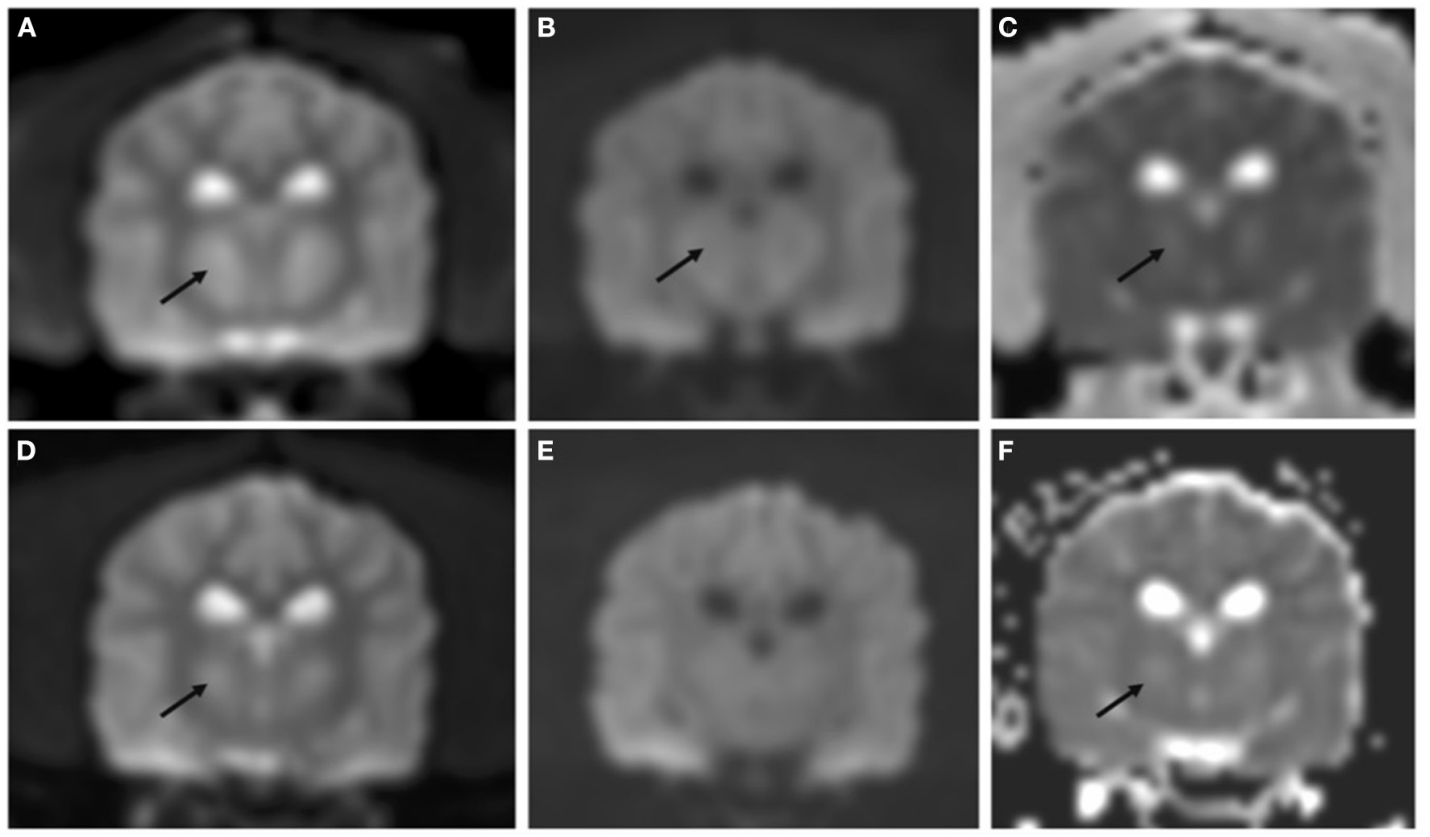
Transverse DWI and ADC map performed on day 4 of hospitalization **(A–C)** and at recheck 130 days later **(D–F)**. Bilateral symmetric hyperintensity on DWI B0 images **(A, D)** and ADC map **(C, F)** is most consistent with T2 shine through. DWI B1000 showed mild hypointensity [arrow in **(B)**] not present at the follow up MRI **(E)**, these findings were most consistent with non-restrictivity.

The patient represented for follow-up imaging four and a half months later. The owner reported that clinically the patient was doing well and had resumed normal activity. A neurologic exam performed by the same clinicians revealed normal neurologic assessment. A brain MRI was repeated, showing improved bilaterally symmetric T2/T2 FLAIR hyperintense/T1 hypo- to isointense non-contrast enhancing lesion of the thalamus, that appeared moderately reduced in size and intensity, and no longer affected the midbrain, which was consistent with resolving myelinolysis. The lesions within the caudate nuclei, rostral cerebral peduncles, and claustrum were less evident (see Figures 1–3).

## 3. Discussion

ODS has been reported in human and canine patients following rapid correction of chronic hyponatremia and is associated with transient or permanent neurologic sequelae (1, 5, 6, 10). Dogs at risk of developing ODS include those that undergo rapid correction of chronic hyponatremia secondary to conditions such as intestinal parasitism, hypoadrenal crisis, chronic effusions, or liver dysfunction (5, 11–13).

The influences of hyponatremia and associated treatments to the brain has been thoroughly reviewed (14). With hyponatremia, the brain takes adaptive measures to ensure the brain cell volume is tightly regulated. Initially, one protective physiologic measure is the shifting of fluid from parenchyma to CSF. The increase in intracranial pressure is postulated to cause hydrostatic water movement from brain tissue into CSF, where it will then be shifted into systemic circulation (15, 16). Additionally, inorganic osmolytes, such as electrolytes, followed by organic osmolytes will be expelled from the brain cells in the minutes to hours and hours to days after the development of hyponatremia, respectively. This reverses and prevents further intracellular movement of water and consequential cerebral edema. When sodium levels rise in a patient with chronic hyponatremia, the opposite processes take place. The brain cells take back the electrolytes and synthesize intracellular proteins and receptors to reuptake the previously secreted organic osmolytes. This process can take up to a week to return to the baseline, and if hyponatremia is corrected too quickly, then these compensatory responses are overwhelmed, and water is allowed to move outside of brain cells, causing shrinkage and apoptosis (15, 16). Furthermore, it was proposed that cytokine release from local inflammation may cause an increase in permeability of the blood brain barrier, allowing additional myelinolytic toxins to enter the privileged CNS, causing damage to the myelin (17). Recent studies suggest an early and central role of astrocyte injury and subsequent blood brain barrier damage. With the development of inflammation, axons may further degenerate, leading to neuronal degeneration (12, 18).

Formerly known as central pontine myelinolysis, this condition was originally thought to solely affect the pons. However, in humans, other locations have demonstrated lesions such as thalamus, cerebellum, lateral geniculate nuclei, and the external and extreme capsules (19). This is consistent with the lesion characteristic and distribution that has thus far been reported in the dog as well (5, 6). Other differentials that could be considered for bilaterally symmetric lesions would include enzyme deficiencies such as the gangliosidoses, toxicities (e.g., bromethalin toxicosis), or nutritional deficiencies (e.g., thiamine deficiency) (20, 21). However, the signalment, history, and lesion distribution differs than that in this condition. For example, dogs affected with gangliosidosis often show signs at a much younger age (22, 23). Thiamine deficiency may also show bilaterally symmetric lesions, however, MRI lesions have been demonstrated in the caudate nuclei, red nuclei, and caudal colliculi (24–26).

Treatment of ODS primarily consists of supportive care and much of the literature focuses on prevention through careful monitoring of sodium levels and slow correction of sodium over several hours to days. However, it is worth noting that experimental animal models showed it is the sodium level changes over 24 h more important than the hourly changes (27, 28). Some novel adjunctive therapies have been reported to help in the treatment of ODS, such as the use of therapeutic plasmapheresis or IVIG, indicating a potential immunological component to the disease process (2, 10, 18). The intravenous administration of myoinositol, a sugar alcohol, suggests improved survival in animal studies, and the use of thiamine supplementation and corticosteroids has also been documented in the treatment of ODS (18, 29). Other pharmacologic interventions described in people include the use of dopamine agonists to aid in the parkinsonian-like symptoms (30). One suggested therapy for treatment in the acute phase of ODS is the use of desmopressin and D5W^17^ to rapidly relower sodium, suggesting that ODS is reversible for a short period of time, despite neurologic signs already occurring (10, 18, 31).

Importantly, in humans with ODS, MRI findings did not prognosticate outcome (2). Laureno et al. performed an experimental study where ODS was induced in dogs, and sequential daily brain MRI were performed up until 6 days. Some dogs showed changes on MRI prior to clinical signs of myelinolysis while other dogs showed clinical signs before changes were detected on imaging (6). These findings are consistent with the unpredictable timeline of clinical signs and imaging abnormalities in humans (2, 6, 10). DWI and ADC map were not supportive of restrictive lesions, unlike what has been reported in some cases of myelinolysis in people (32, 33). This may be due to different timelines in MRI studies; while MRI is typically performed immediately after the onset of clinical signs in people, the MRI study was performed 4 days after the onset of clinical signs in the dog of the present report. It is also possible that the lack of restrictivity may support vasogenic oedema over cytotoxic oedema.

The patient described in this study had a similar progression of clinical signs and was successfully discharged 9 days after the start of neurologic signs, comparably to other case reports in dogs (5, 11). In the described case, adequate pharmacologic support was an essential component of successful management. Midazolam (see text footnote 6), dexmedetomidine (see text footnote 9), and ketamine (see text footnote 12) were all used to facilitate appropriate nursing care, provide nutritional support, and perform blood sampling for monitoring of electrolytes. Midazolam (see text footnote 6) was chosen due to suspected seizure activity and the positive response from the patient. A low dose dexmedetomidine (see text footnote 9) CRI was chosen for its potential muscle relaxation, and ketamine (see text footnote 12) was used for its potential neuroprotective effects of preventing excitotoxic injury.

Lastly, dopamine agonists are chosen in people for its treatment of parkinsonian symptoms such as tremors and bradykinesia. Amantadine (see text footnote 15) is a dopamine agonist and weak antagonist of NMDA-type glutamate receptors. Because of these reports, the patient was started on amantadine (see text footnote 15) at an oral dose of 0.4 mg/kg daily once ketamine (see text footnote 12) infusion was discontinued and oral medications were tolerated. It is suggested that with the use of ketamine (see text footnote 12) and amantadine (see text footnote 15), optimizing dopamine concentration in the synaptic cleft and thus transmission to the post synaptic membrane may be recommended in other suspected or confirmed ODS cases that display tremors (34).

## 4. Concluding remarks

This case report describes the successful management and MRI of a patient with confirmed ODS. Brain MRI was performed at time of diagnosis and at time of follow-up, four and a half months after hospital discharge. This is the first known veterinary case report describing follow-up imaging of a canine after full neurologic recovery of ODS. In humans, patients can have evidence of near to full clinical recovery, yet imaging findings may still be abnormal (7, 35). Another human case report describes full clinical and radiographic recovery 5 months after therapy (10). In this case, the patient had full clinical recovery with evidence of persistent, yet improved myelinolysis. Dogs affected by myelinolysis show very severe clinical signs that may discourage treatment by fear of negative prognosis. However, this report provides evidence that prompt recognition of this pathology by MRI evaluation and intensive management with adequate pharmacologic and nursing support may allow successful improvement of neurological deficits and return to a normal life.

## Data availability statement

The original contributions presented in the study are included in the article/supplementary material, further inquiries can be directed to the corresponding author.

## Ethics statement

Written informed consent was obtained from the participant/patient(s) for the publication of this case report.

## Author contributions

SH and AD wrote the first draft of the manuscript. All authors contributed to conception, design of the manuscript, manuscript revision, read, and approved the submitted version.

## Footnotes

^1^Desoxycorticosterone Pivalate, Elanco Animal Health, Greenfield, IN.

^2^Prednisone, West-ward Pharmaceutical Corp, Eatontown, NJ.

^3^Normosol R^®^, Hospira, Lake Forest, IL.

^4^Dexamethasone SP, Phoenix pharmaceutical, Burlingame, CA.

^5^Butorphanol titrate, Zoetis, Parsippany, NJ.

^6^Midazolam, Fresenius Kabi, Bensenville, IL.

^7^Levetiracetam, Hospira, Lake Forest, IL.

^8^NaCl 0.9%, Dechra, Overland Park, KS.

^9^Dexmedetomidine hydrochloride, Dechra, Overland Park, KS.

^10^Spironolactone, Accord healthcare, Durham, NC.

^11^NaCl 0.45%, Dechra, Overland Park, KS.

^12^Ketamine Hydrochloride, Dechra, Overland Park, KS.

^13^Metoclopramide, Hospira, Lake Forest, IL.

^14^Jevity, Abbott, Abbott Park, IL.

^15^Amantadine, Actavis, Parsippany-Troy Hills, NJ.

^16^Trazodone, Teva, Parsippany, NJ.

^17^D5W, Dextrose with water, Baxter, Deerfield, IL.
